# Secondary physical features in children with FASD

**DOI:** 10.1016/j.ejmg.2023.104890

**Published:** 2023-11-30

**Authors:** Miguel del Campo, Julie A. Kable, Claire D. Coles, Michael Suttie, Christina D. Chambers, Gretchen Bandoli

**Affiliations:** a Division of Dysmorphology and Teratology, Department of Pediatrics, University of California San Diego (UCSD), La Jolla, CA, 92093, USA; b Departments of Psychiatry and Behavioral Sciences and Pediatrics, Emory University School of Medicine, USA; c Department of Obstetrics and Gynaecology, University of Oxford, Oxford, England, UK; d Department of Pediatrics, University of California San Diego, USA

**Keywords:** Fetal alcohol, Dysmorphology, ARND

## Abstract

**Objective:**

The diagnoses included within the umbrella term fetal alcohol spectrum disorders (FASD), are based on the documentation of prenatal alcohol exposure (PAE), growth deficits and a pattern of dysmorphic physical features and neurobehavioral impairments. Although 3 key facial features (short palpebral fissures, a smooth philtrum and a thin vermilion of the upper lip) are the only dysmorphic features taken into account for the diagnosis of Fetal Alcohol Syndrome (FAS) or partial FAS (pFAS), several other features are commonly seen in individuals with these diagnoses. The goals of our study were to determine if some of these secondary physical features also occur more frequently in children with alcohol-related neurodevelopmental disorder (ARND) relative to controls, and if a cluster of these features combined in a dysmorphology score could be used to identify those negatively impacted by PAE but who do not have the cardinal physical features that led to a diagnosis of FAS or pFAS.

**Methods:**

Among 2681 children recruited for the Collaboration on Fetal Alcohol Spectrum Disorders Prevalence (CoFASP) study, 1726 had an FASD or sufficient evidence of PAE having occurred or not in their pregnancy. Children were then categorized into groups using the modified Hoyme diagnostic criteria (FAS (n = 24), pFAS (n = 99) and ARND (n = 87), and No FASD (n = 1516), including those with No FASD and a history of PAE (No FASD/PAE, n = 498) and those with No FASD and no history of PAE (No FASD/No PAE, n = 1018). The frequencies of 26 secondary dysmorphic features were compared among these groups, both individually and combined in non-weighted and weighted dysmorphic scores. Correlations of the total dysmorphic scores with an index of overall cognitive ability were also compared by group status.

**Results:**

Several of these features were significantly more frequent in children with FAS than in those with No FASD diagnosis with or without PAE but not in comparison to those with ARND. The number of features was also significantly higher in the FAS group as compared to all other groups for both weighted and unweighted dysmorphology scores but were not higher in the group with ARND when compared to the groups with No FASD either in the presence or absence of PAE. Although not diagnostic, higher total dysmorphology scores were predictive of lower general cognitive abilities in the group with ARND, suggesting severity of alcohol-related dysmorphology is predictive of severity of alcohol-related neurobehavioral impairment.

**Conclusion:**

Secondary physical features were not more frequent in children with ARND compared to children without an FASD diagnosis but were a marker for lower cognitive function. The use of secondary physical features to support a diagnosis of ARND was not supported in this sample.

## 1. Introduction

Since the original publication on the pattern of malformation of eight offspring of mothers who had an alcohol use disorder, called the Fetal Alcohol Syndrome (FAS) (Jones and Smith, 1973), over 50 years have elapsed. During this time, human studies, cell and animal models have been used to identify the range of manifestations and the nature of the mechanisms by which prenatal alcohol exposure (PAE) adversely impacts the development of the embryo and the fetus. The identification of prenatal alcohol as a teratogen has been well-established (Consensus Statement, 2011). The full range of manifestations that are characteristic of children born after PAE, called the fetal alcohol spectrum disorders (FASD), continues to be debated. Previous research has indicated that diagnostic categories of FASD vary dramatically from one diagnostic scheme to another (Coles et al., 2016), leading to very discordant diagnoses for the same subject within each of the different systems. This disagreement does not facilitate early and universal recognition of FASD, and poses great challenges for comparisons of clinical and research studies in human subjects with PAE. In most diagnostic schemes, four key criteria are used for the definition of all diagnostic categories of FASD. Those are documentation of PAE, growth deficits, a pattern of dysmorphic facial features, and a complex profile of cognitive and behavioral deficits.

Unfortunately, PAE cannot always be confirmed. Mothers may not recall in detail if time has elapsed since pregnancy, may also not tell the truth, and often cannot be accessed to be asked about PAE because the child has been placed in resource families or adopted. In most schemes for the diagnoses of FASD, the presence of the 3 facial features of FAS (short palpebral fissures, a smooth philtrum and a thin vermilion of the upper lip), known as sentinel or cardinal facial features, combined or not with growth deficits, and/or microcephaly/structural brain defects, in the context of neurobehavioral deficits, allow for a diagnosis of FAS or partial FAS (pFAS), even in the absence of confirmation of PAE (Astley, 2013; Coles et al., 2016; Cook et al., 2016; Hoyme et al., 2016). In contrast, the cognitive and behavioral phenotypes of FASD are not considered sufficiently specific to make a diagnosis of Alcohol Related Neurodevelopmental Disorder (ARND) or its equivalents in other diagnostic schemes, when PAE is not confirmed. Further complicating this is that the neurobehavioral characteristics are considered the most complex areas of the spectrum of fetal alcohol effects and there is tremendous variability in operational definitions of these outcomes. Many domains of functioning are not able to be appropriately assessed until children are well past a window of neural plasticity where interventions would be most valuable (Fox et al., 2010). As such, there is motivation to identify biomarkers of PAE that can be reliably measured earlier in life (Bandoli et al., 2022), so interventions and therapies can be applied at a young age. Physical dysmorphic features are easily accessible biomarkers of FAS and pFAS, allowing for a diagnosis in the absence of confirmation of PAE, but historically, this has not been available for those diagnosed with an ARND.

There is evidence that PAE causes additional dysmorphic features that are not incorporated in most diagnostic systems for FASD, but occur more frequently in individuals with FASD in multiple studies (Autti-Ramo et al., 2006; Ervalahti et al., 2007; Feldman et al., 2011; Jones et al., 2010; May et al., 2016; May et al., 2020; May et al., 2021). Some of these features have been identified as being more frequent in FAS and pFAS compared to those without an FASD (Ervalahti et al., 2007; Jones et al., 2010; May et al., 2022; May et al., 2020; May et al., 2021). Higher combined dysmorphology scores have been found to occur more frequently in individuals identified as having FAS or pFAS(Ervalahti et al., 2007; May et al., 2022; May et al., 2020; May et al., 2021) and in some studies to be more frequent in groups categorized as having ARND in comparison to individuals identified as controls (May et al., 2016; May et al., 2021). The first goal of our study was to determine if there was an increased frequency of secondary physical features in each of the FASD diagnostic categories (FAS, pFAS and ARND) relative to those with No FASD either with or without evidence of PAE. We then calculated weighted and non-weighted dysmorphology scores for each of the FASD diagnostic groups and compared the total scores with those with No FASD with or without PAE. Finally, we calculated the correlations of these scores with cognitive abilities in each of the groups of the study. The aim of the study was to identify a cluster of physical features that occurred more frequently within the ARND population that may provide assistance in making this diagnosis earlier in development in the absence of confirmed PAE.

## 2. Material and methods

### 2.1. Study population and sampling

The Collaboration on Fetal Alcohol Spectrum Disorders Prevalence (CoFASP) study used active case-ascertainment methods to identify the prevalence of FASDs in four communities throughout the United States (May et al., 2018). Recruitment took place between 2010 and 2016 at schools. Different sampling methods were used across the four sites and are fully outlined in the original publication (May et al., 2018). Data on 2881 first-grade aged children were then categorized into groups using the 2016 modified Hoyme diagnostic criteria (Hoyme et al., 2016). Participants from the original pool of individuals who were screened were excluded if information about PAE was not collected and there was no evidence of an FASD. As shown in Table 1, of a total of 1726, 1018 participants did not have FASD or PAE (No FASD/No PAE), 498 had PAE but no diagnosis of an FASD (No FASD/PAE), 24 participants had FAS, 99 had pFAS, and 87 had ARND. Details regarding the methods used in making these diagnoses are further elaborated elsewhere (May et al., 2018).

### 2.2. CoFASP study assessments

A common set of maternal questionnaires were used to assess demographic characteristics, details on the pregnancy and health histories. In addition, the four domains assessed in the diagnosis of FASD (PAE, growth, sentinel and secondary dysmorphic features, and neurodevelopmental functioning) were also captured and are described below.

#### 2.2.1. Prenatal alcohol exposure

Trained study staff used a structured interview with mothers or a collateral source to assess PAE for the period before recognition of pregnancy, after recognition of pregnancy, the second trimester and the third trimester. Participants were asked, on average, how much alcohol they consumed in each period, and then asked if they ever drank more than that. For purposes of this study, any alcohol consumption reported in pregnancy was sufficient for the pregnancy to be categorized as positive for PAE.

#### 2.2.2. Growth and dysmorphology

**Fig. 1.**
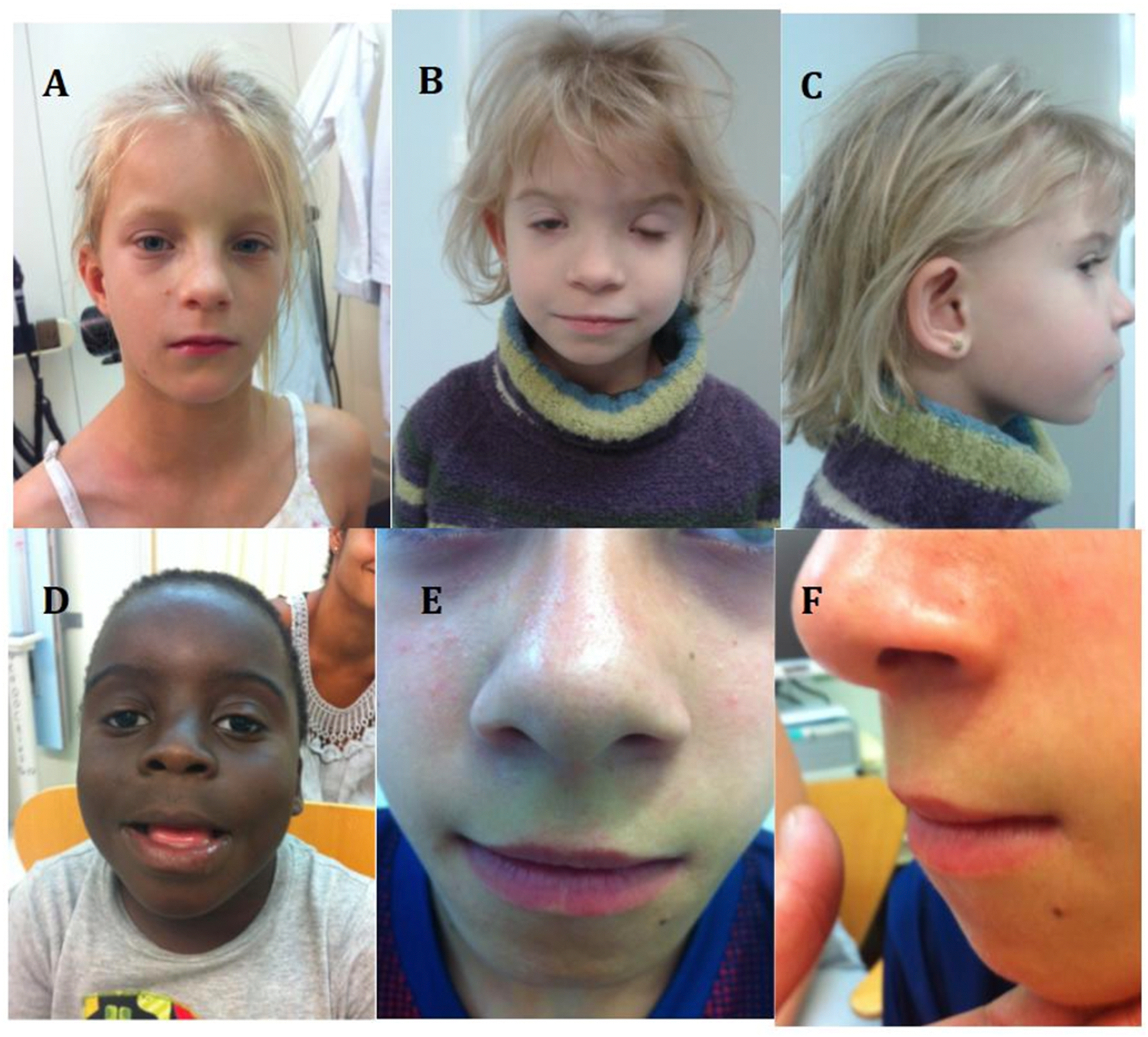
The faces of FASD, showing cardinal and non-cardinal facial features. A. Short palpebral fissures, normal philtrum pillars, narrow and linear vermillion, midface hypoplasia. B Apparent hypertelorism with normal measurements due to the very short palpebral fissures. Prominent epicanthal folds, bilateral ptosis. C. Midface hypoplasia and railroad track ears. D. Mild ptosis, short anteverted nose with long smooth philtrum, fleshy lips. E and F. Frontal and lateral view of the smooth philtrum and linear vermillion border of the upper lip that has lost the characteristic Cupid’s bow configuration. Midface hypoplasia. Reprinted from Eur J Med Genet. 2017 60:55–64. PMID: 27729236.

**Fig. 2.**
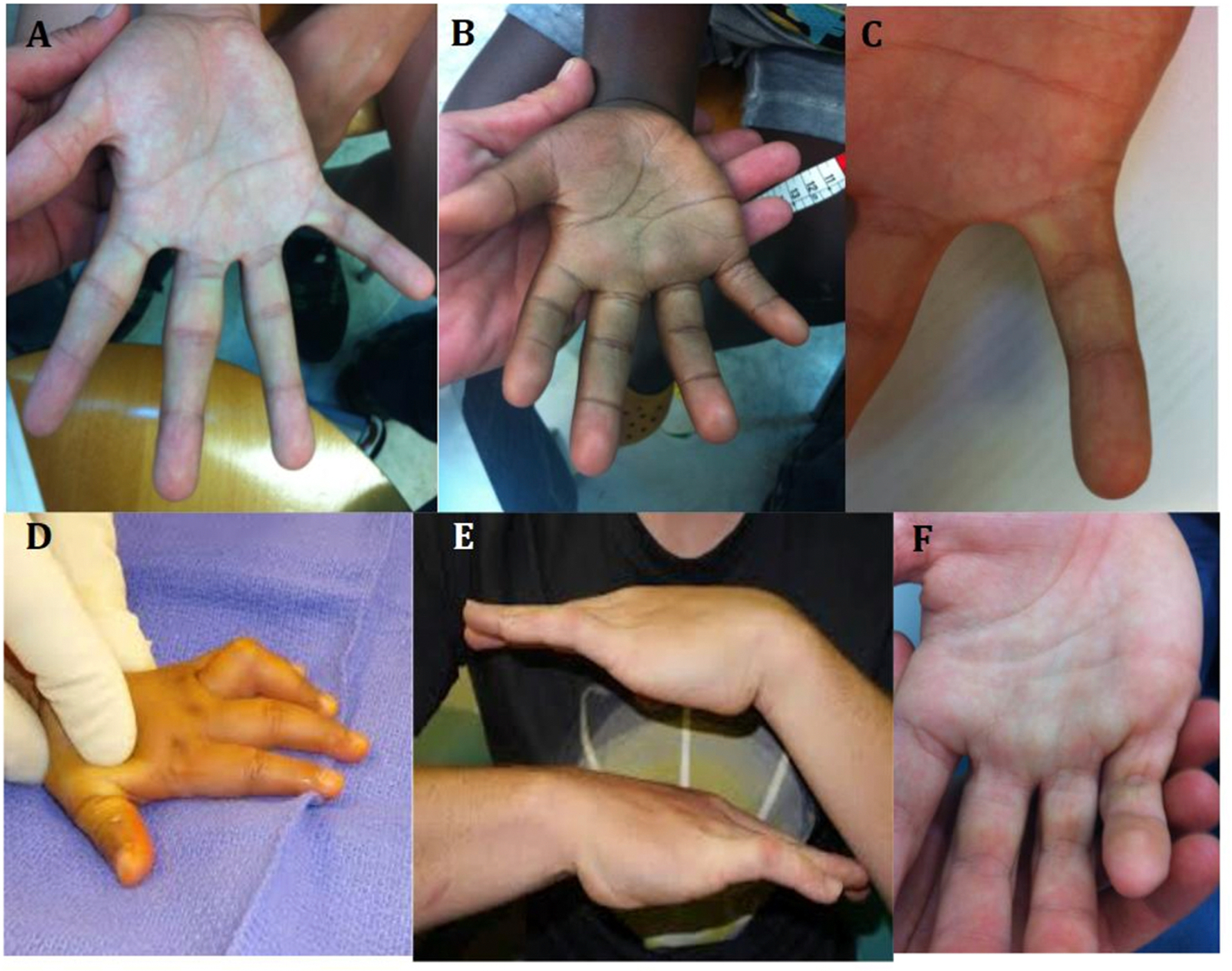
The hands of FASD. A. Hockey stick crease, absent proximal transverse palmar crease, normal thenar crease. B Hockey stick crease, absent proximal transverse palmar crease, absent proximal interphalangeal crease of the 5th finger, limitation to extension of 4th and 5th fingers indicating mild camptodactyly C. Clinodactyly of the 5th finger D. Camptodactyly of the 4th finger. E Bilateral camptodactyly of the 5th fingers. and F. Camptodactyly of fingers 3–4 and 5. Reprinted from Eur J Med Genet. 2017 60:55–64. PMID: 27729236.

A standardized dysmorphology examination was conducted in person by one of several expert pediatric geneticists/dysmorphologists who were blinded to the child’s PAE history. Several of these features are shown in Fig. 1 (facial features) and in Fig. 2 (non-facial features). Measurements of head circumference, height, weight, inner intercanthal distance (aka inner canthal distance ICD), interpupillary distance (IPD), palpebral fissure length (PFL), outer intercanthal distance (aka outer canthal distance OCD), maxillary arc, mandibular arc, and philtrum length were obtained. Centiles for weight and height were calculated from standard growth charts (Kuczmarski et al., 2000) and head circumference centiles were derived from the Nellhaus charts (Nellhaus, 1968). For head circumference, height, weight and PFL, those at or below the 10th percentile were endorsed positive. Endorsements for the presence or absence of the following features or anomalies were also recorded: prognathism, midface hypoplasia, railroad track ears, cupped ears, low set ears, strabismus, ptosis, epicanthal folds, flat nasal bridge, anteverted nose, hypoplastic nails, clinodactyly of the 5th fingers, camptodactyly of one or more fingers, abnormal hand creases (hockey stick crease, single transverse crease, hypoplastic thenar crease, and other), decreased pronation/supination of elbows, knee contractures, hip contractures, other contractures, and hirsutism. Heart murmur and heart defects (ventricular septal defect, atrial septal defect, and other) when identified were also recorded. Also assessed on the exam but not used in our analysis were ratings of abnormal neurologic findings such as hyperactivity, hypertonic muscles, hypotonic muscles, and seizures. A non-weighted score was computed by adding each of the 26 features listed in Table 1 that were endorsed as positive. Separately, a weighted dysmorphology score was calculated based off the score published by Hoyme in 2016 (Hoyme et al., 2016).

#### 2.2.3. Neurodevelopmental functioning

Neurodevelopmental testing was conducted by school psychologists or study psychometrists who were also blind to PAE status and to the results of the physical examination, using a standardized battery to assess neurobehavioral functioning. Details regarding the battery and how it was used to label the diagnostic groups are available in previous publications of this sample (May et al., 2018). For the purposes of this study, the Global Conceptual Ability (GCA) score of the Differential Ability Scales, 2nd edition (DAS-2) (Elliot, 2007), which is a summary score reflecting overall level of intellectual functioning, was used to evaluate relationships between total dysmorphology and cognitive outcome.

#### 2.2.4. Data analyses procedures

Statistical analysis was carried out using SPSS 29. Descriptive statistics (t-tests and chi-squares) were performed on variables related to the 5 groups’ (No FASD/No PAE, No FASD/PAE, ARND, pFAS, FAS) demographic characteristics. Chi-squares were done on endorsement for each of the features sampled and t-tests were done on measurements of features by group status. A total dysmorphology score was then computed by adding positive endorsement for each feature, which was compared by groups status, using generalized analysis of variance. The relationship between the total unweighted dysmorphic score and the DAS-2 GCA was computed for each group using Pearson correlations, which were then compared using the Fisher z transformation statistic. The same was done using the weighted dysmorphology score. Finally, a discriminate function analysis was done to determine if differential weights associated with each feature could differentiate the ARND group from each of the other groups.

## 3. Results

### 3.1. Sample characteristics

The study sample (n = 1726) consisted of 50.8% males and 48.8% females and had a mean age of 7.0 years with a standard deviation of 0.5 years. The racial composition of the sample was 76.0% White, 8.5% Black or African American, 6.7% Biracial, 4.0% Other, 2.6% Unknown, and 2.1% Native American or Native Alaskan.

#### 3.1.1. Group Differences in dysmorphic individual features

Group Differences in Dysmorphic Features are shown in Table 1. The number positively endorsed and the overall percentage by group status for each of the features are presented along with superscripts reflecting statistically distance groups derived from the chi-square analyses. Several of these features such as strabismus, ptosis, 5th finger clinodactyly, camptodactylies, and abnormal palmar creases including hockey stick and single transverse palmar creases, some of which are shown in Figs. 1 and 2, were significantly more frequent in individuals with FAS than in those with both No FASD groups (with or without PAE). As shown in Table 2, there were differences among the FAS/pFAS groups and both No FASD groups for quantitative measures of growth, head size, and sentinel palpebral fissure length and philtrum/vermillion scores but those in the ARND group were similar to those in No FASD groups regardless of the PAE exposure status. Some measurements of decreased facial growth such as maxillary hypoplasia and innercanthal difference were significantly different in FAS relative to all other groups and others such as interpupillary distance and outercanthal distance were different in FAS and pFAS relative to all other groups (ARND, NoFASD/PAE, No FASD/No PAE).

#### 3.1.2. Group Differences in total dysmorphic features

**Fig. 3.**
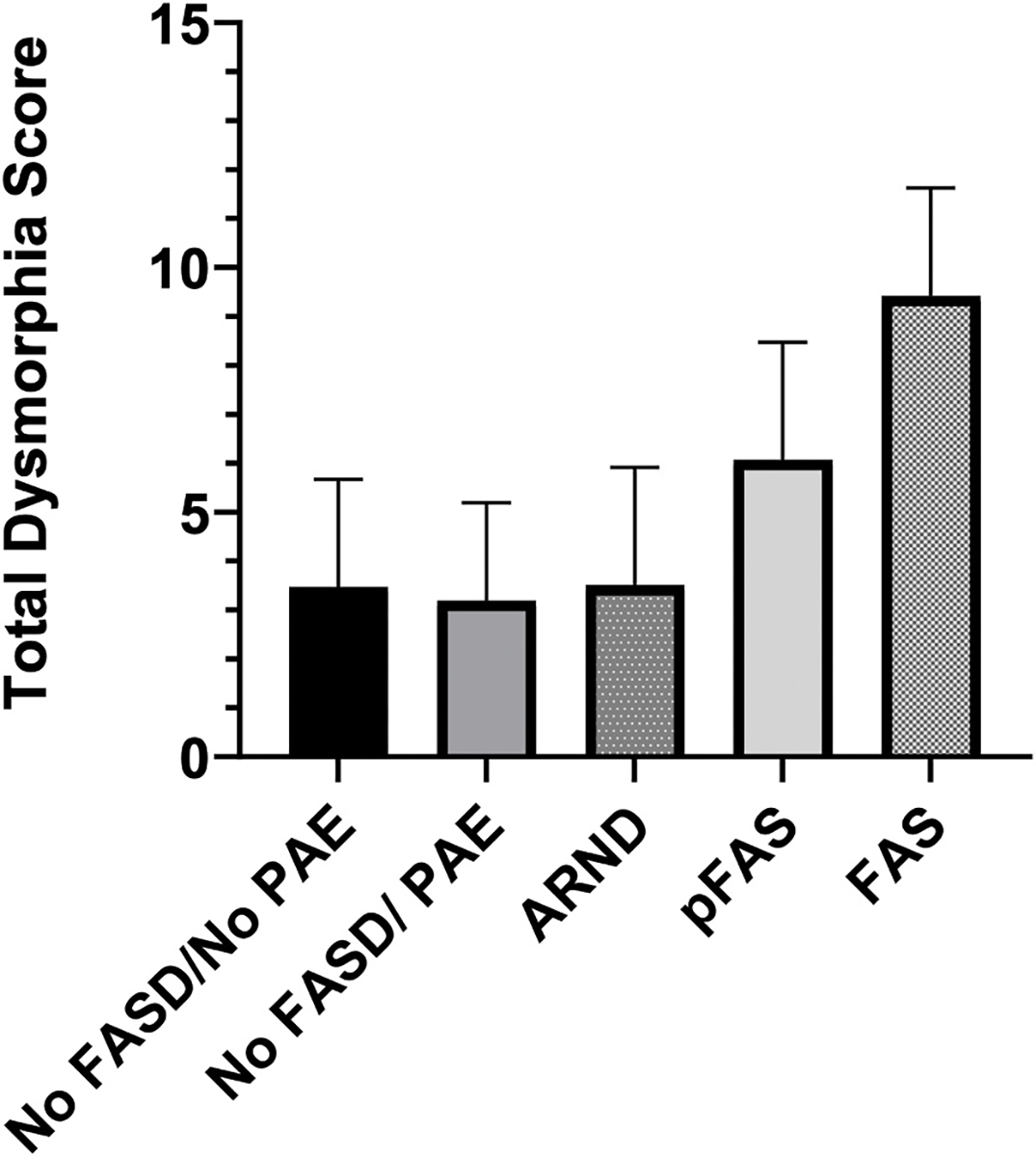
Total Dysmorphic score Bar graphs of the average and 95% confidence interval of the non-weighted total dysmorphic score by group status.

Fig. 3 shows how the groups also differed by non-weighted dysmorphic scores (F (4, 1721) = 56.6, p < .001). The highest dysmorphic score was reported in the FAS group (Mean = 9.42, SD = 2.2) relative to all other groups. The pFAS group (Mean = 6.07, SD = 2.4)\ had the second highest number of features and was significantly higher than those with ARND (Mean = 3.51, SD = 2.4), as compared to those with No FASD/No PAE (Mean = 3.46, SD = 2.2) and those with No FASD/PAE (Mean = 3.19, SD = 2.0). The latter three groups did not differ from each other.

Using the Hoyme weighted score (Hoyme et al., 2016), the groups differed (F (4, 1721) = 126.743, p < .001) in a similar pattern to the unweighted score. The highest weighted dysmorphic score was reported in the FAS group (Mean = 18.7, SD = 4.4). The pFAS group (Mean = 12.7, SD = 3.7) had the second highest weighted score and was significantly higher than those with ARND (Mean = 5.1, SD = 3.9), those with PAE without FASD (Mean = 5.7, SD = 3.9), and those without an FASD and without PAE (Mean = 6.0, SD = 4.1). The latter three groups also did not differ from each other.

#### 3.1.3. Discriminate function analysis of physical features for group status

A discriminant function analysis was done to determine if empirically derived weightings of the score would result in improved group differentiation. As shown in Supplemental Table 1, the ARND group was not able to be distinguished based on secondary dysmorphic features from the combined groups of No FASD/No PAE and No FASD/PAE (df = 31, Wilk’s Lambda = 0.959, χ = 33.245, p < .358, Eigenvalue = 0.043, canonical correlation 0.203) but differed from the combined pFAS/FAS group (df = 29, Wilk’s Lambda = 0.139, χ = 198.076, p < .001, Eigenvalue = 6.177, canonical correlation 0.928) with 100% of the original group correctly classified and 96.6% of the cross-validated sample correctly classified. The pFAS/FAS group significantly differed from the combined groups of No PAE/No FASD and PAE/No FASD (df = 31, Wilk’s Lambda = 0.655, χ = 346.231, p < .001, Eigenvalue = 0.527, canonical correlation 0.587) with 86.6% correctly classified in the original cohort and 84.8% in the cross-validated cohort.

#### 3.1.4. Group Differences in predictive validity of total dysmorphic feature scores

**Fig. 4.**
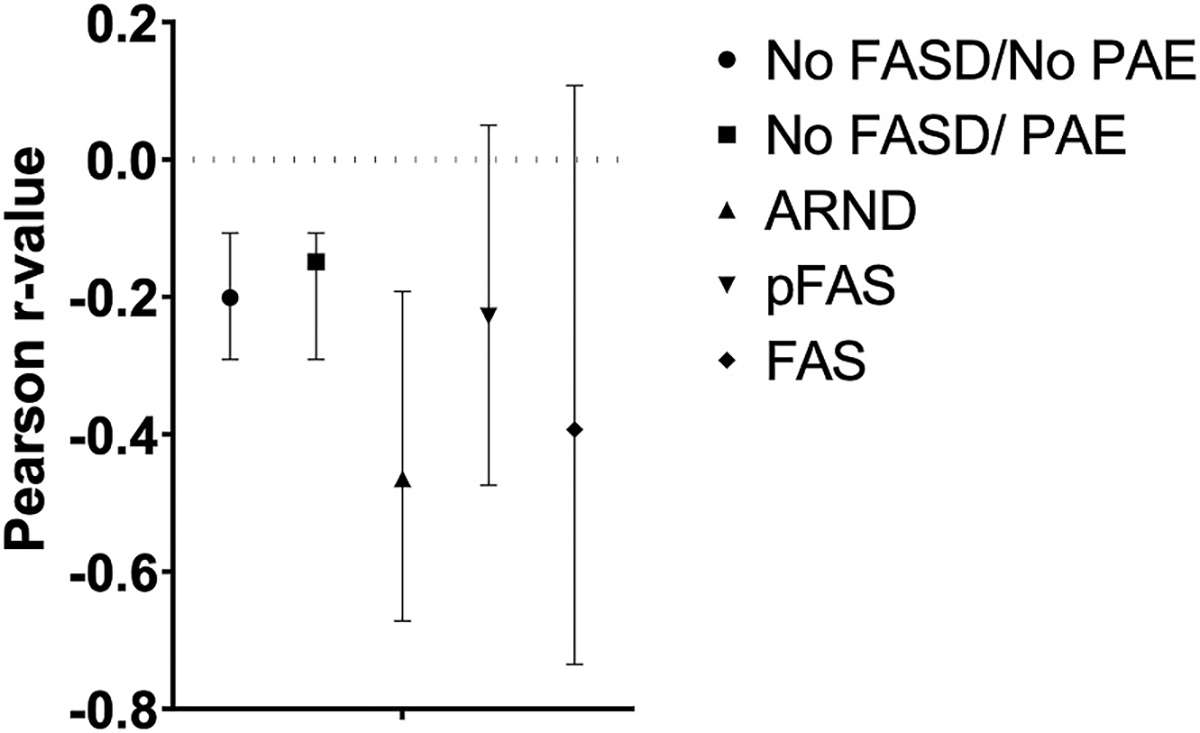
Relationship between Dysmorphic score and DAS-GCA The Pearson correlation value and 95% confidence interval is depicted by group status for the relationship between the Differential Ability Scale, 2nd edition’s General Conceptual Ability score and the total non-weighted dysmorphia score.

Fig. 4 displays the Pearson correlations for the relationship between total unweighted dysmorphic scores and variation in DAS-GCA scores for each group with the 95% CI for each correlation. Those in the ARND group had the strongest negative correlation (−0.465) followed by the FAS group (−0.393). Correlations in the remaining three groups were weaker and did not differ from each other (No FASD/No PAE −0.201, No FASD/PAE −0.149, and pFAS −0.228). Only the group correlations between total dysmorphic scores and DAS-GCA for the ARND and the No FASD/PAE group were statistically different (z = 2.11, p < .0346). A trend was found for this correlation being more negative for the ARND group relative to the No FASD/No PAE group (z = 1.81, p < .07).

## 4. Discussion

The present study was conducted with the following three objectives:

1. Determine if there was an increased frequency of secondary physical features in each of the FASD diagnostic categories relative to those without PAE with a particular focus on identifying features that differentiate those with ARND from those without an FASD either in the presence of PAE or not;
2. Evaluate dysmorphology scores of weighted and non-weighted contributions of cardinal and non-cardinal physical features (i.e., the number and specific type of alcohol-related minor malformations) in all categories of FASD; and
3. Examining whether the dysmorphology scores could be related to measures of intellectual performance in one or all groups.

Using a cohort of 1726 children, who were part of the CoFASP sample (May et al., 2018), we compared the frequency of 26 secondary measurements of facial landmarks and subjective dysmorphic features, among three groups of diagnostic categories of FASD (FAS, pFAS and ARND) and two groups of non-FASD individuals, those with evidence of PAE (No FASD/PAE) and those without evidence of PAE (No FASD/No PAE). Although features were more frequent in FAS in comparison to some or all of the other groups, we did not find that these secondary features were more frequent in the group with ARND when compared to those in either the No FASD groups with and without PAE. The same results applied to weighted and non-weighted dysmorphic scores, which were also higher in FAS but not in ARND relative to the two No FASD groups. Across each analysis attempted, we could not provide evidence that the identification of one or multiple secondary features could support the diagnosis of ARND. We did find that in the ARND group, higher weighted and non-weighted dysmorphology scores were associated with lower cognitive abilities, suggesting that the secondary dysmorphic features may be a marker for more severe alcohol-related neurobehavioral impairment.

The diagnosis of the Fetal Alcohol Syndrome (FAS) is based on the identification of growth deficits, microcephaly, three facial dysmorphic features, and cognitive and behavioral deficits. These 3 facial features, already described in the early publications about FAS (Astley, 2013; Bower et al., 2017; Coles et al., 2016; Cook et al., 2016; Hoyme et al., 2016; Stratton et al., 1996) are considered “sentinel” in all major diagnostic systems for establishing a diagnosis of FAS (Jones et al., 2021). The specificity of their association to PAE and the correlation with the cognitive and behavioral phenotypes of FASD has later been confirmed in multiple studies in different populations and ethnic groups (Astley et al., 2017; May et al., 2016; May et al., 2021). Sufficient evidence has provided confidence for the use of these 3 facial features for the diagnosis of FAS and pFAS, even in the absence of confirmation of PAE, resulting in the most widely used diagnostic systems including the categories FAS and pFAS without confirmed exposure in the FASD spectrum of diagnoses (Astley, 2013; Cook et al., 2016; Hoyme et al., 2016).

In 2004, Streissguth et al. (2004) noted that only ~25% of children affected by in utero exposure to alcohol exhibited physical features. In fact, most individuals seen in the clinic with an FASD carry a diagnosis of ARND and do not have the 3 facial features characteristic of FAS/pFAS, regardless of the diagnostic schemes that are used. Among the 1392 patients followed at the University of Washington, the largest groups of patients diagnosed with an FASD were those diagnosed with static encephalopathy-alcohol exposed (SE-AE) or neurobehavioral disorder-alcohol exposed (NB-AE), the two categories that do not require the sentinel facial features using the 4-digit code (Astley, 2013). In the same sample coded using the Hoyme et al., 2016 guidelines features (Hoyme et al., 2016), ARND, the diagnosis that does not include the physical features, was again the largest group of patients with an FASD (Astley et al., 2017). Similarly, Coles et al. (2016) classified 1581 registered patients at the clinics of Emory University and found that ARND was again the most frequent diagnostic category using 5 different diagnostic schemes. The diagnoses of SE-AE or NB-AE in the 4 digit code, as well as ARND in the Hoyme updated guidelines, can only be made when there is firm evidence of PAE. As neurobehavioral symptoms having significant overlap with other neurodevelopmental phenotypes, including intellectual disability, attention deficit hyperactivity disorder (ADHD), autism spectrum disorder (Kable et al., 2016), and others, identification of specific diagnostic neurobehavioral characteristics that could be used for identification of ARND in the absence of confirmed PAE seems unlikely. In addition, overlap exists between cognitive and behavioral alterations resulting from early trauma exposure that is frequently present in children with PAE. Given incomplete specificity of the neurobehavioral profile and the frequent lack of information on PAE, necessary for the diagnosis of Neurobehavioral disorder related to prenatal alcohol exposure (ND-PAE) (Kable et al., 2016), ARND or its “equivalents” SE-AE and NB-AE, it would be very useful to have some additional physical features that could help diagnose more of the patients that constitute the largest group of individuals with an FASD even in the absence of confirmation of PAE. In the CoFASP sample, the presence of any of these individual features, however, did not help distinguish children with ARND from children without FASD as it did in some previous studies. (May et al., 2016; May et al., 2021). Our results, however, were consistent with those obtained from a subset of the larger CoFASP dataset (May et al., 2022).

Although not relevant to the ARND diagnosis, several specific secondary dysmorphic features were more frequent in children with FAS in the CoFASP sample. Of all the features that were evaluated, strabismus, ptosis of the eyelids, pronosupination of the elbow, 5th finger clinodactyly, camptodactylies, and abnormal palmar creases were more frequent in FAS than in all other groups. The same was true for several facial measurements that can all decrease as a function of a smaller face, such as separation of the eyes (innercanthal distance, interpupillary distance and outercanthal distance) or growth of the midface (maxillary arc). These findings are in agreement with previous studies (Ervalahti et al., 2007; Feldman et al., 2011; Jones et al., 2010; May et al., 2022; May et al., 2020; May et al., 2021).

Although previous efforts had suggested that FASD could be viewed on a continuum of dysmorphology with maximum expression in those categorized as having FAS, lower but increased in pFAS, and potentially still increased in ARND (Del Campo and Jones, 2017), our results were not consistent with this theory. Using a large cohort of participants collected throughout the world, a comprehensive set of physical features were found to occur with higher frequency in individuals with an FAS (Jones et al., 2010). These additional features included a “railroad track” configuration of the ears, ptosis of the eyelids, a “hockey stick” palmar crease, other palmar crease abnormalities, lack of complete extension of one or more digits (camptodactylies), decreased supination/pronation at the elbows, as well as a heart murmur. A subsequent review of multiple studies suggested that these same secondary features were both present at a higher rate in those with FAS but were also more prevalent, to a lesser degree, in those that had some features of FAS, such as growth deficiency, microcephaly or one of the sentinel facial features but who did not fulfill criteria for a diagnosis of FAS/pFAS based on the absence of 2 or more of the 3 sentinel facial features. The findings from th study were supportive of a continuum of dysmorphology being present in those categorized with FAS or pFAS but suggested that this may not extend to those classified with ARND.

The specific individual secondary dysmorphic features found in this sample that were significantly present in those with a diagnosis of FAS were those that have an intimate relationship to the insult to the central nervous system and the visual structures caused by PAE very early in the embryonic developmental period. Abnormal pronosupination of the elbow, 5th finger clinodactyly, camptodactylies, and abnormal palmar creases, all seem to reflect abnormal neuromuscular function leading to abnormal movement early in development. Other features such as strabismus and ptosis also reflect alteration of the visual system and abnormal tone/movements of the extraocular muscles in strabismus and the facial eyelid elevator muscle in ptosis (Tsang et al., 2022).

Whereas different studies have found different sets of individual physical features associated with having an FASD, in almost all cases when those were combined, higher total dysmorphic scores have been found in those categorized as having an FAS or pFAS diagnosis (Autti-Ramo et al., 2006; Ervalahti et al., 2007; May et al., 2022). In some studies, participants with ARND also showed intermediate scores that were clearly higher than the low scores of the non-alcohol exposed children (Ervalahti et al., 2007), suggesting individuals with ARND could be distinguished based on a total dysmorphic scores. In this study, no aggregate value, derived using simple summation or a weighted summation, was able to differentiate the ARND group.

Interestingly, a different approach to quantitative dysmorphology through the study of children with PAE using 3D facial photography (Suttie et al., 2013) found that facial signature graphs that coded volume and distances of the face were well-correlated with the diagnosis of FAS or pFAS. In addition, at least half of the non-syndromic (non-(-FAS/pFAS)) heavily alcohol-exposed children had face signatures consistent with those found in FAS/pFAS groups, leading to the idea that there may be a dysmorphic pattern characteristic of PAE that could serve as a biomarker for those negatively impacted by their PAE. Exposed children with PAE-related facial signatures had lower IQs compared to those with normal facial imaging even in the absence of a diagnosis of FAS/pFAS (Suttie et al., 2013). Quantification of facial dysmorphology that could help identify cases of ARND may be possible using sophisticated facial analysis of 3D images but we did not find that the secondary facial measurements derived from physician measurement and observation could identify those with ARND in our cohort.

Some studies have evaluated the correlation of the dysmorphic features and scores with psychometric measures of cognition (Bandoli et al., 2022; Ervalahti et al., 2007; Suttie et al., 2017). In addition to head circumference and a smooth philtrum., railroad track ears, ptosis, limited prono-supination of elbows and total weighted dysmorphology scores had significant negative correlation with non-verbal IQ in a prevalence study conducted in Italy. Also in Finland, the dysmorphology score correlated with lower IQ and camptodactyly as an individual feature correlated significantly with a measure of verbal intellectual functioning (r (48) = 0.29, *P* < .05) (Ervalahti et al., 2007). The ARND group of this study had the greatest negative correlation with general cognitive abilities, suggesting that dysmorphology scores may be a marker of severity of alcohol-related neurobehavioral impairment based.

The lack of findings of a cluster of secondary features that were predictive of ARND may be influenced by several limitations of the CoFASP study. The number of cases of ARND are a small minority of the cases of FASD, particularly in 3 of the 4 sites, suggesting the study was unable to capture all cases of ARND. Also, multiple examiners were involved in the CoFASP study, raising concerns about the reliability of the assessment of these features, many of which do not have a precise and universal definition (Del Campo and Jones, 2017). In addition, the CoFASP study relied on 7–8 year-old recall of PAE, which may have led to misclassification of the participants based on PAE status.

In conclusion, the data from the CoFASP study does not support the hypothesis that, in the absence of the sentinel facial features of FAS, other physical characteristics alone or combined in dysmorphology scores could help identify individuals with ARND, with or without confirmation of alcohol exposure. We were able to identify, however, that quantitative scores of dysmorphic features have a powerful negative correlation with measures of global cognitive abilities. Further studies are needed to continue to understand how secondary physical features can contribute to improving recognition of FASD across all diagnostic categories. More precise definition of some of these secondary features may facilitate their identification with greater reliability. Studies able to capture the full spectrum of cases of FASD, particularly ARND, may help identify further correlation between the presence of physical features and cognitive and behavioral impairment.

## Supplementary Material

Supplemental material

## Figures and Tables

**Table 1:** Endorsement of dysmorphic features by group status (N, %).

PHYSICAL FEATURE	No FASD/No PAE n = 1018	No FASD/PAE n = 498	ARND n = 87	pFAS n = 99	FAS n = 24	Statistics

HEIGHT ≤10%	119, 11.7^a^	38, 7.7^a^	4, 6.0^a^	21, 26.3^b^	14, 73.7^c^	χ = 97.377, p < .001
WEIGHT ≤10%	95, 9.3^a^	36, 7.2^a^	6, 9.0^a,b^	17, 21.3^b^	13, 68.4^c^	χ = 88.691, p < .001
HEAD CIRCUMFERENCE ≤10%	155, 15.3^a^	64, 13.0^a^	9, 13.4^a,b^	22, 27.5^b^	19, 100^c^	χ = 111.370, p < .001
PALPEBRAL FISSURE LENGTH-LEFT ≤10%	155, 15.3^a^	71, 14.4^a^	7, 8.0^a^	56, 56.6^b^	17, 70.8^b^	χ = 161.176, p < .001
PALPEBRAL FISSURE LENGTH-RIGHT ≤10%	149, 14.7^a^	68, 13.8^a^	8, 9.2^a^	50, 50.5^b^	15, 62.5^b^	χ = 125.547, p < .001
VERMILLION LIPOMETER	186, 18.4^a^	113, 23.0^b^	11, 12.8^a^	83, 83.8^c^	19, 79.2^c^	χ = 257.982, p < .001
PHILTRUM LIPOMETER	201,19.8^a^	82, 16.6^a,b^	3, 4.5^b^	66, 82.5^c^	17, 89.5^c^	χ = 243.449, p < .001
INTERCANTHAL DISTANCE	176, 17.4^a^	81, 16.4^a^	12, 17.9^a^	18, 22.5^a^	6, 31.6^a^	χ = 4.404, p < .354
INTERPUPILLARY DISTANCE	194, 19.2^a^	96, 19.5^a^	12, 17.9^a^	23, 29.1^a^	8, 42.1^a^	χ = 10.605, p < .031
PROGNATHISM	55, 5.5^a^	20, 4.1^a^	7, 4.0^a^	2, 2.5^a^	1,5.3^a^	χ = 6.530, p < .163
MIDFACE HYPOPLASIA	260, 25.9^a^	123, 25.1^a^	15, 22.7^a^	25, 31.3^a^	9, 47.4^a^	χ = 6.260, p < .181
RAILROAD TRACK EARS	88, 8.7^a^	46, 9.4^a^	2, 3.0^a^	7, 8.8^a^	1, 5.3^a^	χ = 3.342, p < .502
EARS CUPPED	13, 1.3^a^	16, 3.3^a^	0, 0.0^a^	3, 3.8^a^	0, 0.0^a^	χ = 9.710, p < .046
EARS LOW SET	6, 0.6^a^	5, 1.0^a^	0, 0.0^a^	1, 1.3^a^	1, 5.6^a^	χ = 6.708, p < .152
STRABISMUS	15, 1.5^a^	11, 2.2^a^	1, 1.5^a,b^	3, 3.8^a,b^	3, 15.8^b^	χ = 21.364, p < .001
PTOSIS	45, 4.5^a^	18, 3.7^a^	3, 4.5^a^	7, 8.9^a,b^	6, 31.6^b^	χ = 34.714, p < .001
EPICANTHAL FOLDS	251, 24.8^a^	87, 17.7^b^	15, 22.4^a,b^	17, 21.3^a,b^	5, 26.3^a,b^	χ = 9.926, p < .042
LOW NASAL BRIDGE	145, 14.4^a^	54, 11.1^a^	12, 17.9^a^	11, 13.8^a^	2, 10.5^a^	χ = 4.403, p < .354
ANTEVERTED NOSE	189, 18.8^a^	82, 16.7^a^	13, 19.4^a^	16, 20.3^a^	3, 15.8^a^	χ = 1.286, p < .864
HYPOPLASTIC NAILS	22, 2.3^a^	4, 0.8^a^	0, 0.0^a^	1, 1.4^a^	1, 5.3^a^	χ = 6.610, p < .158
5 TH FINGER CLINODACTYLY	279, 27.5^a^	159, 32.3^a^	17, 25.8^a^	21, 26.9^a^	10, 52.6^a^	χ = 9.291, p < .054
CAMPTODACTYLY	47, 4.6^a,b^	15, 3.0^b^	6, 9.1^a,b^	3, 3.8^a,b^	3, 15.8^a^	χ = 11.580, p < .021
HAND CREASE-HOCKEY STICK	168, 16.6^a,b^	66, 13.4^b^	6, 9.0^b^	16, 20.3^a,b^	7, 36.8^a^	χ = 12.492, p < .014
HAND CREASE-SINGLE TRAVERSE	22, 2.2^a^	11, 2.2^a^	2, 3.0^a,b^	3, 3.8^a,b^	3, 15.8^b^	χ = 15.287, p < .004
HAND CREASE-HYPOPLASTIC THENAR	2, 0.2^a^	0, 0.0^a^	0, 0.0^a,b^	0, 0.0^a,b^	1, 5.3^b^	χ = 28.564, p < .001
HAND CREASE-OTHER ABERRANT	49, 4.8^a^	17, 3.4^a^	2, 3.0^a^	5, 6.4^a^	0, 0.0^a^	χ = 3.458, p < .484
ARMS-DECREASED PRONATION/SUPINATION	18, 1.8^a^	8, 1.6^a^	0, 0.0^a^	3, 3.8^a^	1, 5.3^a^	χ = 4.464, p < .347
KNEE CONTRACTURES	0, 0.0^a^	0, 0.0^a^	0, 0.0^a^	0, 0.0^a^	0, 0.0^a^	n/a
LEGS/FEET OTHER CONTRACTURES	0, 0.0^a^	1, 0.2a	0, 0.0^a^	0, 0.0^a^	0, 0.0^a^	χ = 2.186, p < .701
HIP CONTRACTURES	0, 0.0^a^	0, 0.0^a^	0, 0.0^a^	0, 0.0^a^	0, 0.0^a^	n/a
HIRSUTISM	27, 3.0^a^	16, 3.4^a^	0, 0.0^a^	5, 6.8^a^	0, 0.0^a^	χ = 6.113, p < .191
HEART MURMUR	0, 0.0^a^	3, 0.6^a,b^	0, 0.0^a,b^	1, 1.3^a,b^	1, 5.3^b^	χ = 22.824, p < .001
NEUROLOGICAL SIGNIFICANT EXAM	5, 0.8^a^	1, 0.3^a^	1, 2.4^a^	0, 0.0^a^	0, 0.0^a^	χ = 3.799, p < .434

Note: FASD = fetal alcohol spectrum disorders, PAE = prenatal alcohol exposure, FAS = fetal alcohol syndrome PFAS = partial FAS, ARND = alcohol-related neurodevelopmental disorder.

**Table 2:** Differences in measurements of growth and facial features by group status (mean, standard deviation).

SYMPTOM	No FASD/No PAE n = 1018	No FASD/PAE n = 498	ARND n = 87	pFAS n = 99	FAS n = 24	Statistics

HEIGHT PERCENTILE	48.88 (29.2)^a^	53.05 (28.4)^a^	53.10 (28.2)^a^	32.77 (26.3)^b^	8.54 (10.1)^c^	F (4, 1717) = 22.917, p < .001
WEIGHT PERCENTILE	53.99 (29.7)^a^	55.11 (28.7)^a^	56.72 (30.4)^a^	36.87 (28.3)^b^	11.00 (12.9)^c^	F (4, 1718) = 21.195, p < .001
OCCIPITOFRONTAL PERCENTILE	48.68 (30.8)^a^	55.72 (31.5)^a^	50.53 (33.3)^a^	33.37 (26.3)^b^	4.46 (3.4)^c^	F (4, 1712) = 25.047, p < .001
BODY MASS INDEX PERCENTILE	57.39 (29.6)^a^	55.91 (29.0)^a^	57.92 (32.6)^a^	47.42 (30.6)^a^	32.08 (29.0)^b^	F (4, 1717) = 6.639, p < .001
PHILTRUM LENGTH PERCENTILE	48.78 (29.8)^ab^	43.14 (29.7)^ab^	49.49 (31.2)^ab^	53.67 (33.5)^a^	35.69 (24.5)^b^	F (4, 1102) = 3.063, p < .016
PHILTRUM SCORE	3.00 (.68)^a^	2.93 (.71)^a^	2.83 (.72)^a^	3.84 (.55)^b^	4.00 (.59)^b^	F (4, 1710) = 51.417, p < .001
VERMILLION SCORE	2.93 (.70)^a^	3.00 (.71)^a^	2.83 (.67)^a^	3.90 (.46)^b^	3.88 (.54)^b^	F (4, 1709) = 55.639, p < .001
PALPEBRAL FISSURE LENGTH LEFT PERCENTILE	27.67 (16.6)^a^	27.07 (15.4)^a^	30.02 (15.0)^a^	14.22 (14.3)^b^	10.25 (12.0)^b^	F (4, 1709) = 23.229, p < .001
PALPEBRAL FISSURE LENGTH RIGHT PERCENTILE	28.18 (16.6)^a^	27.57 (15.5)^a^	30.86 (14.9)^a^	15.28 (14.2)^b^	10.21 (10.1)^b^	F (4, 1710) = 22.528, p < .001
PROPORTION OF PFL OF ICD	.8523 (.08)^a^	.8633 (.08))^ab^	.8498 (.09))^ab^	.8193 (.08)^b^	.8458 (.08)^ab^	F (4, 1709) = 6.079, p < .001
MAXILLARY ARC	24.82 (1.2)^a^	24.64 (1.1)^a^	24.88 (1.4)^a^	24.45 (1.0)^a^	23.28 (1.0)^b^	F (4, 1707) = 13.186, p < .001
MANDIBULAR ARC	25.95 (1.4)	25.77 (1.3)	26.05 (1.6)	25.53 (1.2)	24.04 (1.3)	F (4, 1706) = 14.037, p < .001
INNERCANTHAL DISTANCE PERCENTILE	53.97 (25.6)^a^	50.47 (23.2)^a^	57.91 (26.3)^a^	51.15 (25.9)^a^	39.0 (21.5)^b^	F (4, 1710) = 4.498, p < .001
OUTERCANTHAL DISTANCE PERCENTILE	32.73 (21.5)^a^	28.59 (19.1)^a^	33.83 (20.0)^a^	25.47 (19.9)^b^	16.63 (12.8)^b^	F (4, 1706) = 8.603, p < .020
INTERPUPILLARY DISTANCE PERCENTILE	55.74 (26.2)^a^	53.17 (25.5)^a^	56.23 (25.5)^a^	48.22 (26.9)^b^	38.13 (22.2)^b^	F (4, 1704) = 4.795, p < .001

Note: FASD = fetal alcohol spectrum disorders, PAE = prenatal alcohol exposure, FAS = fetal alcohol syndrome PFAS = partial FAS, ARND = alcohol-related neurodevelopmental disorder.

## Data Availability

Data will be made available on request.
