# Supplementary material for: Secondary physical features in children with FASD
Source: Eur J Med Genet. Author manuscript; Available in PMC 2025 Apr 4. (PMC11970374; doi:10.1016/j.ejmg.2023.104890)
Supplement: Supplemental material [file NIHMS2059669-supplement-Supplemental_material.docx]

| Symptom | ARND  vs  No PAE/No FASD, PAE/No FASD | ARND  vs  pFAS/FAS | pFAS/ FAS  vs  No PAE/No FASD, PAE/No FASD |
| --- | --- | --- | --- |
| Vermillion Lipometer | -.192 | .923 | .473 |
| Philtrum Lipometer | -.085 | 1.177 | .501 |
| Palpebral Fissure Length-Left | -.431 | 1.072 | .449 |
| Palpebral Fissure Length-Right | -.028 | -.062 | -.066 |
| Height | .103 | -.082 | .313 |
| Weight | .397 | -.087 | .121 |
| Occipitofrontal Circumference | .121 | -.061 | .138 |
| Intercanthal Distance | -.088 | .092 | -.018 |
| Interpupillary Distance | .113 | -.042 | -.098 |
| Prognathism | .503 | -.260 | -.007 |
| Midface Hypoplasia | -.041 | .058 | -.044 |
| Railroad Track Ears | -.036 | .245 | .025 |
| Ears Cupped | -.133 | .007 | .050 |
| Ears Low Set | -.087 | .ns | -.038 |
| Strabismus | .038 | .063 | .019 |
| Ptosis | .141 | .059 | .066 |
| Epicanthal Folds | -.021 | -.078 | .034 |
| Flat Nasal Bridge | .351 | -.453 | -.008 |
| Anteverted Nose | -.194 | .140 | .035 |
| Hypoplastic Nails | .003 | -.162 | .034 |
| 5^th^ Finger Clinodactyly | .102 | -.006 | .008 |
| 5^th^ Finger Camptodactyly | -.010 | .016 | .045 |
| Other Dysmorphia | .115 | -.028 | -.024 |
| Hand Crease-Hockey Stick | .048 | -.016 | .068 |
| Hand Crease-Single Traverse | -.047 | -.006 | .035 |
| Hand Crease-Hypoplastic Thenar | -.118 | ns | -.111 |
| Hand Crease-Other Aberrant | .034 | -.145 | .033 |
| Arms-Decreased Pronation /Supination | -.095 | .105 | .086 |
| Knee Contractures | ns | ns | ns |
| Legs/Feet Other Contractures | ns | ns | ns |
| Hip Contractures | ns | ns | ns |
| Hirsute | -.297 | .020 | .043 |
| Heart Murmur | .079 | .036 | -.032 |
| Heart | -.100 | .031 | .122 |
| FASD=fetal alcohol spectrum disorders, PAE=prenatal alcohol exposure, FAS=fetal alcohol syndrome PFAS=partial FAS, ARND=alcohol-related neurodevelopmental disorder | | | |

Supplemental Table 1. Standardized Discriminative Weight Estimates Associated with each dysmorphic feature
